# Stable distribution of reciprocity motives in a population

**DOI:** 10.1038/s41598-020-74818-y

**Published:** 2020-10-23

**Authors:** Jeroen M. van Baar, Felix H. Klaassen, Filippo Ricci, Luke J. Chang, Alan G. Sanfey

**Affiliations:** 1 Department of Cognitive, Linguistic, and Psychological Sciences, Brown University, Providence, RI USA; 2 Donders Institute for Brain, Cognition, and Behavior, Radboud University, Nijmegen, The Netherlands; 3 Utrecht School of Economics, Utrecht University, Utrecht, The Netherlands; 4 Department of Psychological and Brain Sciences, Dartmouth College, Hanover, NH USA; 5 Behavioral Science Institute, Radboud University, Nijmegen, The Netherlands

**Keywords:** Human behaviour, Psychology

## Abstract

Evolutionary models show that human cooperation can arise through direct reciprocity relationships. However, it remains unclear which psychological mechanisms proximally motivate individuals to reciprocate. Recent evidence suggests that the psychological motives for choosing to reciprocate trust differ between individuals, which raises the question whether these differences have a stable distribution in a population or are rather an artifact of the experimental task. Here, we combine data from three independent trust game studies to find that the relative prevalence of different reciprocity motives is highly stable across participant samples. Furthermore, the distribution of motives is relatively unaffected by changes to the salient features of the experimental paradigm. Finally, the motive classification assigned by our computational modeling analysis corresponds to the participants’ own subjective experience of their psychological decision process, and no existing models of social preference can account for the observed individual differences in reciprocity motives. These findings support the view that reciprocal decision-making is not just regulated by individual differences in 'pro-social’ versus ‘pro-self’ tendencies, but also by trait-like differences across several alternative pro-social motives, whose distribution in a population is stable.

## Introduction

Cooperation is central to the evolutionary success of humans and many other organisms^1^. One important mechanism for cooperation is reciprocity, whereby individuals take turns incurring a loss to benefit the other^2,3^. Several social preferences have been proposed as motivations for reciprocal decisions. For example, individuals may act reciprocally to avoid unequal payoffs between themselves and interaction partners (inequity aversion)^4–7^, or to avoid feelings of guilt associated with disappointing others (guilt aversion)^8,9^. Recent evidence shows that different people are motivated by qualitatively different sets of these reciprocity motives, with some individuals exhibiting context-dependent motives such as moral opportunism^10^. This raises the question whether the distribution of reciprocity motives across people in a population is stable and predictable, or whether it may instead change with time and decision task. This is an important question because individual differences in motives for social decision-making—and beliefs about others’ motives—can determine whether an intervention to increase prosocial action backfires or succeeds^11–15^.

To address this question, we test two hypotheses about the distribution of reciprocity motives observed across three studies. The *population property hypothesis* holds that the experimentally observed distribution of reciprocity motives reflects a stable property of the sampled population, similar to the distribution of trait-like features of persons such as agreeableness, aggression, and moral concerns^16–18^. If this hypothesis is true, we should find a similar prevalence of reciprocity motives every time we sample from the same population, even if the framing of the task is changed. In contrast, the *strategy salience hypothesis* posits that an experimentally observed distribution of reciprocity motives is primarily a consequence of contextual and framing effects. This prediction is grounded in a wealth of literature documenting changes in the distribution of social decisions across experimental tasks and decision contexts (see Ref.^19^ for a meta-analysis), in part due to the influence of framing on beliefs and social norms^20–22^. If this alternative hypothesis is true, we should observe significant variation in the distribution of reciprocity motives across experimental samples, particularly when changing the salient features of the task. Importantly, these two hypotheses are not mutually exclusive. It is likely that both person-dependent and context-dependent influences jointly determine reciprocity motives, reflecting the person-situation debate^23^ that has revealed both person- and situation-dependent determinants of social choice behavior^24–26^. However, given the recent doubts about generalizability of behavior in laboratory tasks^19^, finding a stable distribution of motives across experiments and participant samples (as per the population property hypothesis) would be particularly informative.

Prior economic game experiments only provide limited evidence toward either of these hypotheses. First, although many studies have characterized pro-social versus pro-self tendencies in observable social behavior (e.g.^27–30^), researchers often remain agnostic about the psychological motives that underlie these behavioral tendencies. Second, existing work has typically not repeatedly probed a population to examine the stability of social choice patterns across multiple samples. Instead, the stability of social decision-making is usually evaluated by carrying out several different laboratory and field experiments using the same sample of participants (e.g.^19^). Although we acknowledge the informativeness of this approach, it is not without its potential pitfalls. For instance, participants may either attempt to be consistent in their behavior across tasks due to a preference for consistency^31^ or might balance prosocial behavior in one task with selfish behavior in another^32^. A mix of such sequential effects would undermine measurement of the stability of social decision-making motives. In this work, therefore, we do not compare behavior in the same people across tasks, but instead compare behavior on the same task across three sets of participants sampled from the same population. This allows us to test whether individual differences in reciprocity motives are stably distributed in a population or are sensitive to the framing of a task, which speaks to the trait-like nature of reciprocity motives without testing the same individuals multiple times.

We use variants of the Trust Game^33^ to detect participants' reciprocity motives including inequity aversion and guilt aversion. In a regular Trust Game, Player 1 (the Investor) invests any proportion of a $10 endowment in Player 2 (the Trustee), retaining the remainder. The investment is multiplied × 4 before being sent to the Trustee, who then freely decides how much of the multiplied amount to return to the Investor. Trustees commonly return about 50% of the multiplied investment^34^, which is in line with both inequity aversion^4,5^ and guilt aversion^8^ and thus introduces a confound in the interpretation of Trustee decisions^10,35^. To resolve this confound, the Hidden Multiplier Trust Game used here multiplies the investment by × 2 or × 6 instead of × 4 on half the trials^10^. Although the Investor believes that the multiplier remains × 4, the Trustee is aware of the true multiplier and of the Investor’s ignorance, and can thus take advantage of the information asymmetry between the players. This paradigm reveals pronounced individual differences in Trustees’ motivations, even though their behavior is indistinguishable in a regular Trust Game. Computational modeling shows that about 40% are mainly inequity-averse (always returning half the multiplied investment), 10% are mainly guilt-averse (always returning what the Investor expects, insensitive to the changing multiplier), 40% are morally opportunistic (advantageously acting inequity-averse in × 2 and guilt-averse in × 6), and 10% are greedy (returning little to no money)^10^.

Here, we compare three independent datasets of Hidden Multiplier Trust Game data, collected years apart, to test the stability of this distribution of reciprocity motives. Moreover, we manipulate two properties of the Hidden Multiplier Trust Game to further probe whether the distribution is a stable feature of the population or an artifact of the experimental task. First, the *task incentives* of the HMTG place a large financial pressure on guilt-averse Trustees because these players need to sacrifice all their game tokens in the × 2 condition to meet the Investor’s expectations. In study 1 we relax this financial pressure by administering the HMTG twice to the same participants, once with the original × 2/× 4/× 6 multipliers and once using multipliers × 4/× 6/× 8 (see “Methods”), testing the stability of participants’ strategies under changing incentives. Second, the *salient strategy* in the HMTG is inequity aversion, since this strategy’s decisions change between the task conditions while guilt-averse choices remain the same. In study 2, therefore, we presented new participants with a ‘flipped’ version of the task, the False-Belief Multiplier Trust Game (FBMTG), where the Investor believes the multiplier is × 2, × 4, or × 6, but the Trustee learns that the true multiplier is always × 4. Here guilt aversion is the salient strategy as the task explicitly manipulates the expectations of the Investor. We test whether this raises the prevalence of guilt aversion and lowers the prevalence of inequity aversion, as predicted by the strategy salience hypothesis.

Across both studies, we employ an integrative model of social preferences to quantitatively map the reciprocity motives of our participants. In this Moral Strategy model (Eq. 1^10^), two free parameters (Θ and Φ) determine the influence of three sources of utility—payoff (*π*), guilt, and inequity—on decisions. For a detailed description of the model, see “Methods”.

1 $$U_{2} \left( {S_{2} } \right) = \Theta *\pi_{2} {-}\left( {1{-}\Theta } \right)*\min \left( {Guilt_{2} + \Phi ,Inequity_{2} {-}\Phi } \right)$$

## Results

### Computational modeling

**Figure 1:**
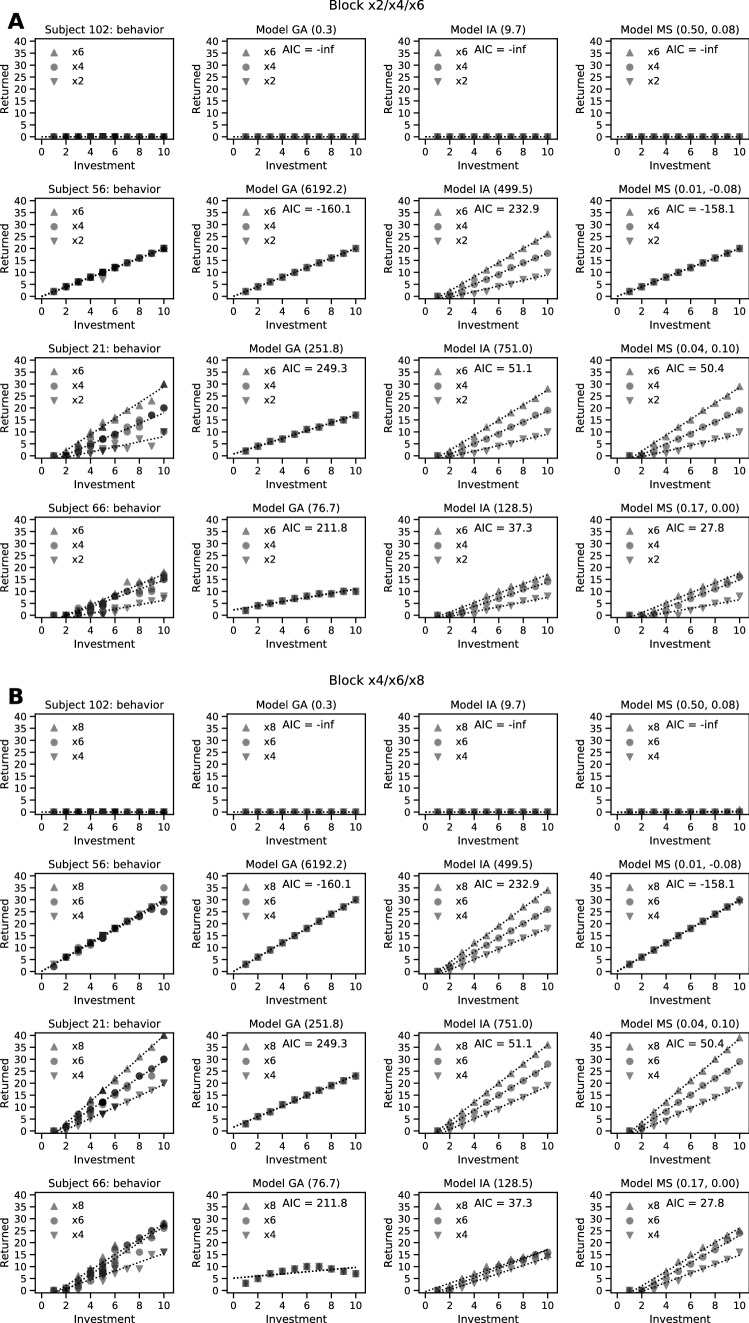
Left panels: task behavior for four example participants in study 1, block × 2/× 4/× 6 (**A**) and × 4/× 6/× 8 (**B**). Other panels: Model predictions for the GA, IA, and MS (moral strategy) models, fitted to these behavioral data. This posterior predictive check reveals that the MS model was best at capturing the four predicted behavioral strategies that are exemplified by these four example participants. The superior fit of the MS model is confirmed quantitatively in formal model comparison (see Fig. 2A).

Different participants showed markedly different patterns of reciprocity behavior in the Hidden Multiplier Trust Game (illustrated in Fig. 1 for study 1). To formally test whether these differences reflected between-subject differences in reciprocity motives rather than noise around a single decision strategy shared across all participants, we compared the model fit of the three unitary social preference models (greed, guilt aversion, and inequity aversion) to that of our Moral Strategy model^10^. This latter approach integrates these reciprocity motives into one model and allows the individual weights to vary across participants.

**Figure 2:**
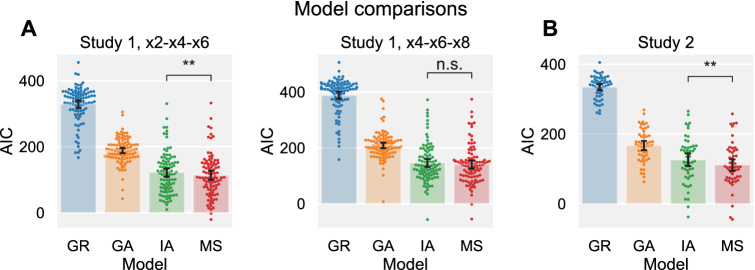
Model comparisons for the moral strategy (MS), inequity aversion (IA), guilt aversion (GA) and greed (GR) models. (**A**) Model comparisons across the two contexts of study 1. (**B**) Model comparisons in study 2. AIC: Akaike Information Criterion. **p < 0.01 (uncorrected). Error bars represent bootstrapped 95% confidence intervals.

In study 1, we fit all four models to the participants’ behavior separately in each of the two HMTG blocks (2/× 4/× 6 and × 4/× 6/× 8). In the × 2/× 4/× 6 block, model fit was significantly better for the Moral Strategy (MS) model than for the greed model, the guilt aversion model, and the inequity aversion model (paired-samples t-test on AIC between MS model and best other model, i.e. MS vs. IA: *t*(93) = −2.65, *p* = 0.0096; Fig. 2A). Although the improved model fit for the MS model relative to IA was not significant in the × 4/× 6/× 8 context (MS vs. IA: *t*(93) = −1.31, *p* = 0.194), mean model performance across the two contexts was significantly better for MS (paired-samples t-test on mean subject AICs across contexts, IA vs. MS: *t*(93) = 2.07, *p* = 0.042).

In study 2, model fit was significantly better for the Moral Strategy model compared to the unitary preference models (i.e., greed, guilt aversion, and inequity aversion models) (Fig. 2B) (paired-samples t-test on AIC between IA and MS models: *t*(52) = 3.31, *p* = 0.002). We thus replicate our earlier results^10^, showing that a set of qualitatively different reciprocity motives can better capture the behavior in the HMTG than any unitary social preference model by itself, even when penalizing for increased model complexity. Posterior predictive checks on four representative participants (Fig. 1) confirm that the Moral Strategy model better captured the key differences between participants than did either of the guilt aversion and inequity aversion models.

### Determining participants’ reciprocity motives

One notable advantage of the Moral Strategy model is that by fitting this model to each participant’s behavior, we obtained a pair of parameters (theta and phi) that best captured that particular participant’s reciprocity motives. We can then classify each participant’s moral strategy based on their position in the theta–phi parameter space of the model. For each of the four theoretically predicted reciprocity motives (greed, GR; guilt aversion, GA; inequity aversion, IA; moral opportunism, MO), a zone can be identified where this moral strategy is best represented, by use of the model-driven clustering procedure described in^10^. In brief, this clustering method consists of first simulating task behavior across equidistant theta–phi coordinates in the parameter space, and then using a hierarchical clustering algorithm to group these simulations based on pairwise similarity in simulated task behavior (for details see “Methods”). Of note, this method does not rely on any participant data, and can therefore be flexibly applied across experiments with different task parameters (e.g. different sets of multipliers).

**Figure 3:**
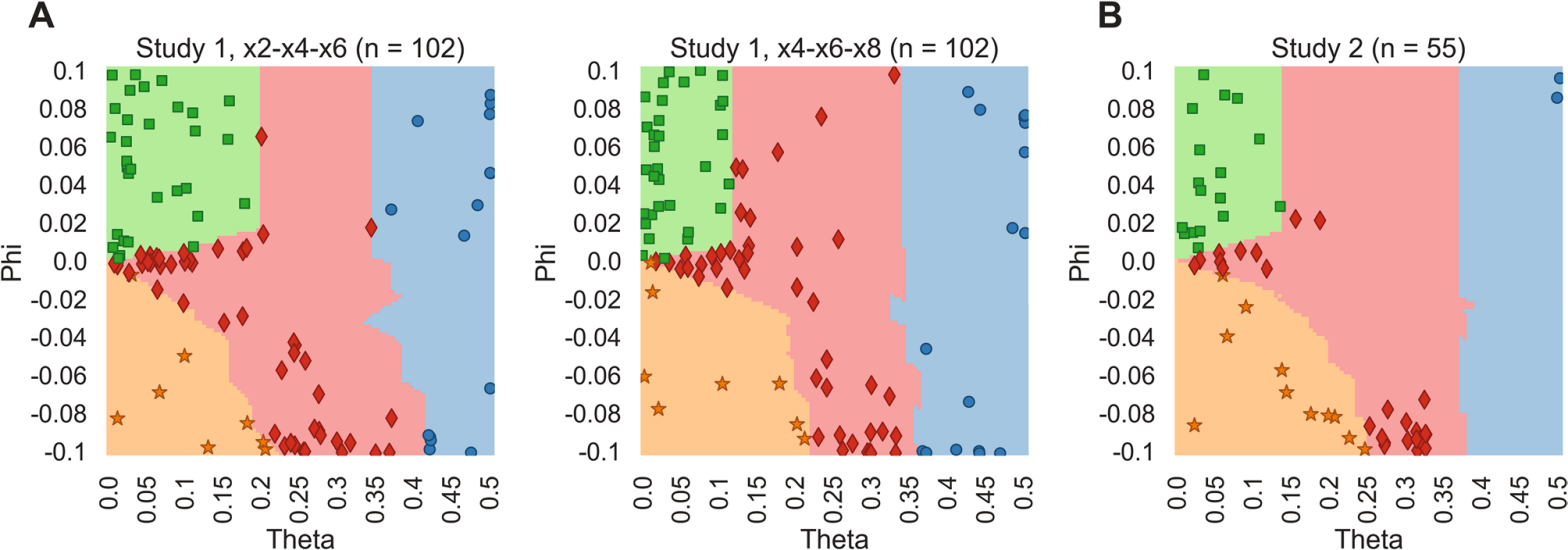
Model parameter space in study 1 (**A**) and 2 (**B**). The space contains the four hypothesized reciprocity motives at different zones of parameter combinations (colored zones), with best-fitting parameters added in for each participant (scattered points). Circles: greed; stars: guilt aversion; squares: inequity aversion; diamonds: moral opportunism.

Figure 3 shows the position of each participant in the theta–phi model parameter space across both studies and sets of multipliers, overlaid on the colored zones that represent the four reciprocity motives. Participants are labeled by moral strategy based on their position in one of the four strategy zones, and participant behavior for each cluster is plotted in Supplemental Fig. 1 as a qualitative confirmation of clustering accuracy.

### Can financial pressure drive guilt-averse participants to moral opportunism?

**Figure 4:**
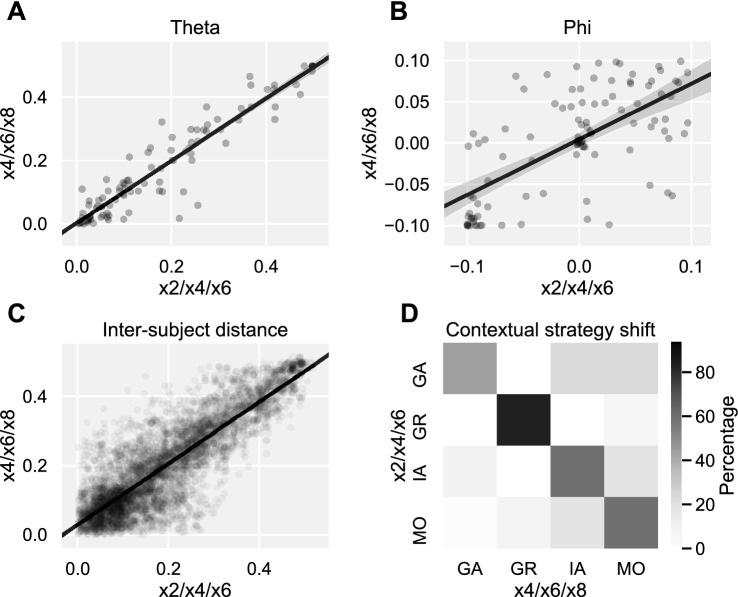
Moral strategy is stable across the × 2/× 4/× 6 and × 4/× 6/× 8 context of study 1. (**A**, **B**) Test–retest reliability of model parameters theta and phi between the two contexts is high. (**C**) Test–retest reliability of the inter-subject distance in the model parameter space is high. Inter-subject distance captures the geometry of similarity between participants in theta-phi space, which is useful since small changes in parameter values are expected between the × 2/× 4/× 6 and × 4/× 6/× 8 blocks due to the changing multiplier magnitude. (**D**) Categorical shifts in moral strategy between the two contexts are rare (highest values on the diagonal).

To examine the stability of participants’ reciprocity motives across blocks in study 1, we quantified the test–retest reliability of model parameters theta and phi. It should be noted that small changes in these parameters are expected across blocks, since the boundary between strategies in the parameter space changes slightly as a function of multiplier set (× 2/× 4/× 6 vs. × 4/× 6/× 8, see Fig. 3A,B). Therefore, we also computed test–retest reliability of the distance between each pair of participants in the theta–phi parameter space. If participants employ stable motives across blocks, these inter-participant distances should remain stable even if small shifts in theta/phi occur for each participant. For test–retest reliability, we computed the Pearson correlation for theta, phi, and inter-participant distance between blocks × 2/× 4/× 6 and × 4/× 6/× 8. All three metrics yielded very high reliability across contexts (theta: *r* = 0.95, *p* < 0.001; phi: *r* = 0.66, *p* < 0.001; inter-subject distance: *r* = 0.86, *p* < 0.001; Fig. 4A–C), which suggests that participant behavior was relatively insensitive to the multiplier manipulation, which supports the population property hypothesis but not the strategy salience hypothesis.

As a more precise test of the strategy salience hypothesis, we hypothesized that the financial pressure of the × 2 condition of the HMTG could push otherwise guilt-averse players towards a strategy of moral opportunism. If this interpretation were true, we would expect participants who were morally opportunistic in the × 2/× 4/× 6 block to exhibit a guilt-averse behavior pattern in × 4/× 6/× 8. We tested this prediction using a Stuart–Maxwell test for paired (repeated-measures) frequency sets, which revealed no significantly different distribution of strategies between the two multiplier contexts (*X*^2^(3) = 1.48, *p* = 0.69; Fig. 4D). A more specific Stuart–Maxwell test for switching between MO and GA revealed no significant change in relative frequency of these two strategies between the two contexts (*X*^2^(1) = 0.33, *p* = 0.56). On the contrary, as Fig. 4D shows, most participants were consistent in their moral strategy between blocks × 2/× 4/× 6 and × 4/× 6/× 8 (the highest values lie on the diagonal), and only 2.1% of moral opportunists in × 2/× 4/× 6 were guilt-averse in × 4/× 6/× 8. This suggests that the financial pressure imposed by the × 2 condition did not specifically shape the distribution of reciprocity motives in the × 2/× 4/× 6 context, which supports the population property hypothesis over the strategy salience hypothesis.

### Does task salience affect moral strategy?

**Figure 5:**
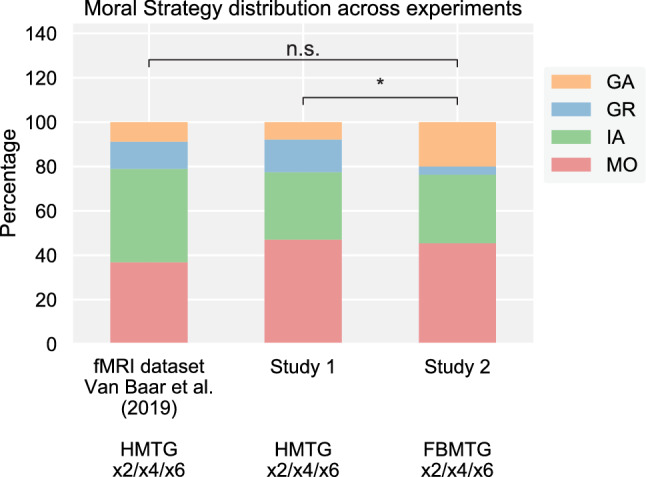
The relative prevalence of the four reciprocity motives in prior work (fMRI dataset; Van Baar et al.^10^), study 1 (× 2/× 4/× 6 block), and study 2. HMTG, Hidden Multiplier Trust Game; FBMTG, False-Belief Multiplier Trust Game. *Difference in moral strategy distribution is significant at *p* < 0.05; n.s. not significant.

Study 2 was designed to make guilt aversion more salient than in study 1 by manipulating the expectations of the Investors, and should conversely make inequity aversion less salient than in study 1. To test these predictions of the strategy salience hypothesis we compared the relative prevalence of the four reciprocity motives between study 2 and the × 2/× 4/× 6 block of study 1 using a chi-square test for independent samples. The results are plotted in Fig. 5, alongside the distribution of reciprocity motives found in our prior work^10^. Overall, the relative prevalence of the four strategies was significantly different between study 1 and study 2 (*X*^2^(3) = 8.43, *p* = 0.038). At first glance, this effect appears to be driven by an increased prevalence of GA in study 2 as compared to study 1 (Fig. 5): in study 2 GA is no longer the least popular moral strategy, but the second least popular. However, this increase in GA prevalence was not accompanied by a decrease in IA prevalence, as IA was about equally common in study 1 as in study 2. Indeed, when testing the between-study difference in relative prevalence of each pairwise combination of two strategies, the only significant change was in the relative prevalence of GA and GR (*X*^2^(1) = 6.40, *p* (uncorrected) = 0.011), which may be a false positive since this effect does not hold when Bonferroni-correcting for multiple pairwise tests (*p* (corrected) = 0.069). IA and MO were just as dominant in study 2 as in study 1. Given this mixed evidence, we additionally compared the strategy prevalence in study 2 to that of our prior work on the HMTG^10^. Between these studies, no significant change in strategy prevalence was found (*X*^2^(3) = 6.54, *p* = 0.088), and none of the pairwise prevalence shifts were significant (all *p* (uncorrected) > 0.05). Taken together, these results provide some support for the strategy salience hypothesis, but also highlight the striking consistency of reciprocity motives across three different datasets, collected at different times, in different experimental settings (fMRI, behavioral), and, crucially, with entirely different salient features in the experimental paradigms.

### Construct validity of moral strategy

The combined results of study 1 and 2 suggest that the observed distribution of reciprocity motives is a stable property of the population from which we sampled. This interpretation raises the question whether the moral strategy labels we apply to participants in fact correspond to the participant’s own experience of their psychological decision process (i.e., construct validity). We tested this by asking all participants in the two studies to self-report on how they made their decisions in the HMTG and FBMTG, suggesting four potential strategies and leaving room for other strategies in an open-ended answer box. To maximize our statistical power for this follow-up analysis we pooled data across studies 1 and 2. To avoid including participants who were unstable in their moral strategy, we kept only the 74 participants from study 1 who exhibited the same moral strategy category across the two multiplier blocks. The total pooled *n* was therefore 74 + 55 = 129.

**Figure 6:**
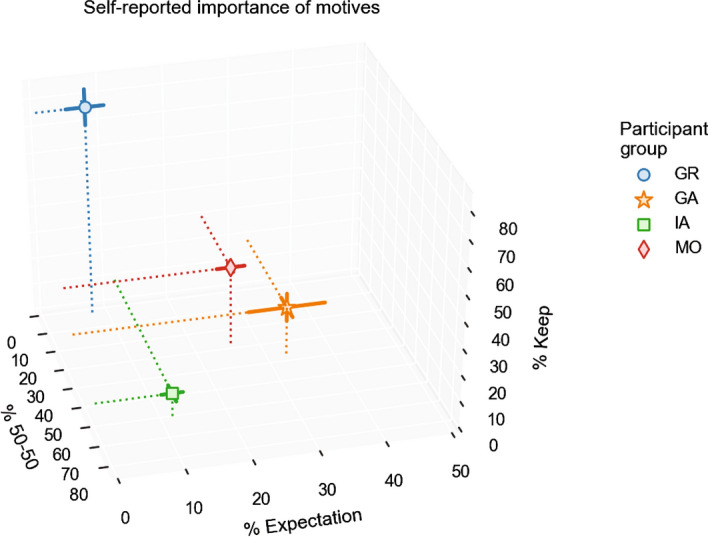
Self-reported importance of the three most prominent decision strategies for participants in the four model-derived moral strategy groups. Points are mean over group; solid lines represent standard error of the mean; dotted lines are perpendicular projections from the mean points onto the axes.

As predicted, there were significant differences in the weight assigned by the four groups (GR, GA, IA, MO) to the first three of the suggested strategies (‘Keep’: *F*(3,125) = 52.51, *p* < 0.001; ‘50–50’: *F*(3,125) = 33.49, *p* < 0.001; ‘Expectation’: *F*(3,125) = 13.63, *p* < 0.001) (Fig. 6). To better understand these findings, we tested whether each of these three suggested strategies was self-reported as most important by the associated moral strategy group. This was indeed the case: Inequity-averse participants assigned significantly higher weight to ‘50–50’ than the other three groups (IA-GA: *p* = 0.006; all other IA pairs *p* < 0.001); greedy participants assigned significantly higher weight to ‘Keep’ than the other three groups (all *p* < 0.001); and guilt-averse participants rated the ‘Expectation’ strategy as more important than all other groups, although this effect was not significant for GA-MO (*p* = 0.16; all other GA pairs *p* < 0.01). Taken together, these results lend construct validity to our model-based strategy interpretations, suggesting that we have appropriately labeled the reciprocity motives present in our participants.

### Determinants of moral strategy

Can any other commonly used measures of social decision strategy account for the individual differences reported in this paper? To answer this question, we compared the model-derived reciprocity motives to participants’ scores on three other measures: Social Value Orientation (SVO;^36^), dispositional greed^37^, and trait guilt^38^. If model parameter theta (the ‘greed parameter’) accurately captures the greed aspect of moral strategy, we would predict that, across participants, this parameter is negatively correlated with the social value orientation (SVO) score, which is known to predict prosociality in a range of laboratory tasks^36,39^. In line with this expectation, we found a strong and significant negative correlation between theta and SVO (*r* = −0.68, *p* < 0.001), such that higher SVO scores were related to lower theta values. We also found a significant, though weaker, positive correlation between theta and dispositional greed (DG) (*r* = 0.21, *p* = 0.017). Theta was not correlated to trait guilt, as expected. Due to a significant negative correlation between phi and theta (*r* = −0.38, *p* < 0.001), raw phi values also correlated with SVO, but in the opposite direction from the theta relationships (phi with SVO: *r* = 0.28, *p* = 0.0011). To control for this confound, we residualized phi with respect to theta (i.e. took the residual of the regression of phi onto theta); residualized phi was not significantly correlated with either SVO (*p* = 0.74) or DG (*p* = 0.22). Surprisingly, residualized phi was also not associated with trait guilt (*p* = 0.61). These results suggest that previously proposed measures of social preference (social value orientation and greed) capture the same trait as our model’s theta parameter, but that parameter phi captures previously unexplained variance in social choice behavior. Therefore, Hidden Multiplier games offer a novel approach to both examine actual behavior in an interactive setting, as well as offering a unique assay for directly measuring the motives underlying reciprocity behavior.

## Discussion

In this work, we demonstrate that (a) different people follow qualitatively different reciprocity motives when making reciprocity decisions in a modified Trust Game, (b) these motives are stable over time within a person within an experiment, (c) the distribution of motives across datasets is stable, and (d) the salience of a specific reciprocity motive only weakly affected the prevalence of that motive in the data. We further illustrate that the reciprocity motives, derived from computational utility models grounded in economic theory, align with participants’ own experience of their psychological decision process, which lends construct validity to our model. Taken together, these findings support the population property hypothesis over the strategy salience hypothesis. Our results thus suggest that social decision-making is not just regulated by individual differences in 'pro-social’ versus ‘pro-self’ tendencies, but also by trait-like differences across several alternative pro-social motives, whose distribution in a population is relatively stable.

Our findings contribute to the person-situation debate in social psychology by showing that different salient features of a Trust Game (i.e. context and framing effects) do not strongly affect the observed distribution of reciprocity motives. Given that all participants within one experimental sample were presented with the same stimuli and framing, the observed individual differences in motives must have resulted from differences at the person level. Importantly, these motives had highly similar distributions across experiments, suggesting that they are represented in a trait-like manner in the population from which we sampled, which therefore makes them relatively robust to changes in the decision context. Future research may evaluate how these distributions of motives are maintained within a population. One possibility is that social norms regulate the motives that come into play when individuals make social decisions^40^. Another is that reciprocity motives are yoked to stable traits such as interpersonal attachment style^27^ and political preference^17,41^, which together shape an individual's 'moral phenotype' that defines their social choice behavior. Similarly, the stability of the distribution of reciprocity motives could itself be a feature of the particular population under study—in this case, a student population from the Netherlands. Comparative cross-cultural research could test these alternative explanations, and taking an intergenerational or developmental perspective could illuminate potential roles of cultural evolution, phenotypic plasticity^42^, and socio-ecological perturbations such as resource scarcity in shaping the distribution of decision motives in a population. It is important to understand how distributions of decision motives arise in a population, as these motives could potentially play important roles in whether government interventions backfire or succeed^12,13,43^.

Our findings further suggest that stability in social decision-making may be found not at the level of observable behavior, but rather at the level of underlying motives. For instance, in the Hidden Multiplier Trust Game, guilt-averse participants sometimes keep nearly all of the money they receive from the Investor, but at other times retain almost nothing. Despite these large behavioral differences, the underlying motive is nonetheless stable: return what the Investor expects to receive. Such paradoxical behavior is also found in moral opportunists, who switch between guilt- and inequity-averse patterns of behavior. Since this strategy is nevertheless highly consistent, and actually avoids greed, the underlying motive here may also be stable: always adhere to a moral rule, without caring much about which rule this actually is from trial to trial. This evidence shines new light on the low behavioral correlations often observed across experimental tasks such as economic games. It is possible that low behavioral correlations may result from high reliability in underlying motives. For instance, one participant may be generous in a laboratory dictator game out of concern for reputation, while another generous participant may be motivated by simple altruistic preferences. The behavior of these two participants might then be diametrically opposed when they partake in an unobserved a real-world decision. This distinction could also potentially explain low behavioral correlations between observations in the lab and in the field^19^. This possibility is rarely evaluated in the literature, as it is not clear how to detect underlying motives in most economic games such as the ultimatum game and prisoner's dilemma. Our work provides a starting point with an experimental task that can detect the motives underlying reciprocity decisions in a trust game with good construct validity.

The generalizability of this work is limited in several ways. First, we do not evaluate the test–retest reliability of the observed reciprocity motives we report here, as we do not test the same sample of participants multiple times. We believe our approach of instead sampling from the same population multiple times has merit, as it circumvents the aforementioned pitfalls associated with repeatedly testing the same individuals. Nevertheless, further research comparing the decision motives of a set of participants across time would be very informative for determining precisely how stable and trait-like decision motives are. Second, the implications of our findings are thus far limited to laboratory trust games. Although this is so by design, as our approach leverages presenting the same task multiple times to different groups of people, it would be insightful to test whether the reciprocity motives observed here generalize across tasks. At present, such research is challenging as there are few ‘standard’ tasks that allow researchers to infer several distinct motives underlying observable behavior (with some notable exceptions^22^). Development of these tasks would be very valuable, and could potentially benefit from false-belief manipulations as in the HMTG and FBMTG.

We observed a weak effect of task salience on moral strategy. This is somewhat surprising, as demand effects in social psychology are common^44,45^ and suggest that participants should track the most clearly manipulated component of a task in their behavior. Nevertheless, the increase in prevalence of GA relative to GR between studies 1 and 2 may indicate that demand effects may play a modest role in some motives for reciprocity, while other reciprocity motives (e.g. inequity aversion) are always salient, regardless of task manipulation.

Future work could improve our understanding of the inequity aversion and guilt aversion motives described here. As a further specification of inequity aversion, it is possible that individual differences in advantageous versus disadvantageous inequity aversion contribute to differences in social decision-making^46,47^. As for guilt aversion, it is puzzling that the individual difference measure of ‘trait guilt’ (part of the Guilt Inventory as described by^38^) did not predict guilt-averse preferences in the HMTG. This lack of relationship may be understood by investigating what is meant by ‘guilt’ in these two different contexts. The Guilt Inventory questionnaire items loading on ‘trait guilt’ relate to the guilt one feels individually when thinking back to a past misdeed (e.g. one item reads “I have made a lot of mistakes in my life”), as well as guilt related to disappointing authority (e.g. “My parents were very strict with me”). Guilt aversion, as defined in our study and by Ref.^8^, instead describes an interpersonal emotion^48^ that results from disappointing an equal interaction partner. These two types of guilt thus point to distinct psychological experiences. Importantly, feelings of ‘guilt’ as operationalized in the Guilt Inventory could follow from behaving unfairly just as well as from disappointing an interaction partner, and so it remains to be seen when and why such feelings are experienced by decision-makers in economic games. Further research could contribute to understanding guilt aversion by developing a questionnaire measure for interpersonal guilt, by measuring feelings of guilt during the HMTG, and by clarifying the relationship between different types of guilt on the one hand and regret and shame on the other.

Taken together, our findings caution against interpreting social choice behavior through the lens of a single motive, as individual differences in decision motives play an underappreciated role in social decision-making. By formally modeling different motives and teasing them apart using innovative experimental tasks, we can begin to better understand idiosyncratic laboratory behavior as well as the social decisions that define our societies.

## Methods

### Study 1

#### Participants

Both studies were carried out in accordance with the Declaration of Helsinki and were approved by the local ethics review board (CMO Arnhem-Nijmegen, The Netherlands). In study 1, 104 adult participants were recruited from the Nijmegen student population using an online recruitment tool. People were excluded from participation if they had taken part in an experiment on the Hidden Multiplier Trust Game before, if they were students of economics, if they were not native speakers of Dutch, if they were pregnant, and/or if they suffered from claustrophobia. 2 participants were excluded from analysis due to not believing the task instructions, leaving a total of 102 participants (32 men and 70 women; mean age 22.3 ± 2.7 years).

#### Task

Both studies in the present paper use the Hidden Multiplier Trust Game (HMTG;^10^). In each trial of this game, an anonymous Investor gets the chance to invest any number of 10 game tokens with an anonymous Trustee, retaining the remainder. These are all ‘single-shot’ games, that is, the Investor and Trustee interact only once. As in a standard Trust Game^33^, the invested amount is increased by a multiplier before being transferred to the Trustee. The Trustee then gets the chance to return any number of tokens from the multiplied amount to the Investor, but, importantly, does not have to do so. The tokens are redeemed for money at the end of the experiment (exchange rate: €0.40 per token).

In the standard Trust Game, the multiplier is × 4, and both the Investor and the Trustee have full knowledge of this. In the Hidden Multiplier Trust Game, however, the multiplier is sometimes × 2 (25% of rounds) or × 6 (25%) instead. Crucially, the Investor believes the multiplier is always × 4, whereas the Trustee knows both about the multiplier used, as well as about the Investor’s ignorance as to the actual multiplier. In the HMTG, therefore, the Trustee sometimes has more or fewer tokens than the Investor believes, and the expectations of the Investor are uncoupled from the true amount of money received by the Trustee.

Whereas guilt aversion and inequity aversion models make the same behavioral predictions in the classic Trust Game, these predictions diverge in the HMTG. Specifically, a guilt-averse Trustee, motivated to satisfy the expectations of the Investor^8^, should send the same amount of money back to the Investor in each of the three multiplier conditions, as the Investor’s expectations do not change across these conditions. An inequity-averse Trustee, on the other hand, is motivated to provide an even split of all available tokens in the game^4,5^, and should therefore return more money in the × 6 condition than in the × 4 condition, and more in × 4 than in × 2, that is, they should be motived by the actual amount of tokens they possess. A morally opportunistic Trustee combines these two decision strategies adaptively, returning enough to satisfy the Investor’s expectations in the × 6 condition, while creating an even split in the × 2 condition^10^. A greedy Trustee, finally, should not send back any tokens. These four ‘reciprocity motives’ are formalized in the computational model below.

In study 1, participants played two blocks of the Hidden Multiplier Trust Game^10^, each with 80 trials. One of the two blocks of 80 rounds of the HMTG was played using the multiplier set of [× 2, × 4, × 6], where the Investor believed the multiplier was always × 4. The other block was played with multiplier set [× 4, × 6, × 8], and here the participants were instructed that the Investors always believed the multiplier was × 6. The order of the two blocks was counterbalanced across participants.

#### Procedure

The experiment consisted of a single session. Upon arrival at the laboratory, each participant was seated in a closed-off behavioral lab space, where they were screened for participation, were informed about the study, and gave written informed consent. Next, they received written general instructions about the standard Trust Game. To avoid biasing game behavior, the Trust Game was always referred to as ‘Investment game’, the Investor as ‘player A’, and the Trustee as ‘player B’. The multiplier in these standard instructions was the *Investor-believed multiplier* of the block in which the participant started (i.e. × 4 for × 2/× 4/× 6 block first, and × 6 for× 4/× 6/× 8 block first). Importantly, these instructions contained no information about the ‘hidden multiplier’ aspect of the task, and were meant to inform the participants about the general structure of this game. In these instructions, the participants were asked how many of 10 game tokens they would themselves invest in an anonymous Trustee, if they were the Investor (and the multiplier was the base multiplier of × 4 or × 6). They were asked for consent to use this investment in a future experiment on the Trust Game, and were informed that if their investment was used in the future, they would receive additional payment based on the amount sent back to them by the future Trustee (‘player B’). We additionally asked for permission to take their portrait photo, which was to be used alongside their investment in the potential future experiment. All these questions were added to the instructions to ensure that the investments made here by the participants were sincere, and to ensure that the participants believed that they were interacting with real investors (i.e. people who participated at an earlier time). In reality, the participants played with pre-recorded investment data from a single-shot Trust Game with multiplier × 4^10^. This instruction step additionally allowed us to generate a new set of true investments for future experimental use.

After reading the general Trust Game instructions, the participants read additional instructions pertaining to the ‘hidden multiplier’ element of the Hidden Multiplier Trust Game. Here, we informed them that the Investors they would encounter always believed that the multiplier was fixed (at × 4 for participants who started with the × 2/× 4/× 6 multiplier block, and at × 6 for × 4/× 6/× 8), but that the actual multiplier would sometimes be lower (× 2 or × 4) or higher (× 6 or × 8). After testing the participants on their understanding of these instructions in a short quiz, they played 80 single-shot, anonymous rounds of the Hidden Multiplier Trust Game, always in the role of Trustee, in the context they were assigned to first (× 2/× 4/× 6 or × 4/× 6/× 8).

On each round, the Investor’s participant number was presented alongside a blurred photo of a face, which was added to strengthen the belief that the Trustees were playing with real Investors. The photos were taken from the Radboud Faces Database^49^ and blurred heavily in Matlab to ensure that participants could not recognize the gender or identity of the faces. On the next screen, the participants saw how many tokens the Investor had invested in them, and by which factor this investment was multiplied. The participants were also reminded that the Investor always believed the multiplier was × 4. On the final screen, the participants could indicate how many tokens from the multiplied amount they wanted to return to the Investor; crucially, they could also choose to keep it all. There was a short break after 40 trials.

After playing 80 rounds in the first context, the participants were allowed to take a self-paced break while remaining in the behavioral lab space, and received instructions for the second context. These short instructions highlighted the fact that the multiplier set would change, and that the new Investors for this second context always believed the multiplier was × 6 (in × 4/× 6/× 8) or × 4 (in × 2/× 4/× 6). Thus, all participants played 80 rounds of the HMTG twice, once in × 2/× 4/× 6 and × 4/× 6/× 8, with context order counterbalanced between participants, and they were informed that they played with a unique anonymous Investor on each of the 160 rounds of this experiment. The investment sets were identical between the two contexts. The participants were informed that one trial out of the 160 would be randomly selected to be paid out both to them and to the corresponding Investor.

Since the multiplier in the HMTG determines with how many tokens the Trustee gets to play, it also determines the behavioral predictions of the inequity aversion model: under the raised multiplier condition (× 6 or × 8) the amount sent back to the Investor by the Trustee ought to be higher than under the base multiplier (× 4/× 6) or the lowered multiplier (× 2/× 4). Since the Investor always believes the multiplier to take the base value (× 4 in × 2/× 4/× 6 and × 6 in × 4/× 6/× 8), however, the predictions of the guilt aversion model are identical across multiplier conditions. This allows us to deduce from each Trustee’s behavior which moral strategy they followed in their reciprocity decisions (see Computational modeling).

#### Additional measures

After finishing the HMTG, participants completed a computerized version of the social value orientation (SVO^36^) task, which was based on the ‘slider measure’ version of the SVO^39^. Then, they completed the dispositional greed scale^37^, which measures greediness as a personality trait, and the guilt inventory^38^, which yields an individual difference measure of general sensitivity to guilt (trait guilt). Finally, they answered several questions about their own decision-making strategy in the HMTG, dividing 100 points over various descriptions of strategies:

- Inequity aversion (“I wanted to divide the total number of tokens evenly over the Investor and myself”)
- Guilt aversion (“I wanted to give the number of tokens that the Investor expected to receive”)
- Greed (“I wanted to keep as much of the investment as possible for myself”)
- Altruism (“I wanted to return as much as possible of the investment”)
- Other (“Other, describe and indicate number of points”)

#### Computational modeling

We used the Moral Strategy model^10^ to infer the Trustees’ motives based on their reciprocity behavior in the HMTG. This model (Eq. 1) describes the utility derived by the Trustee from their strategy *S*_2_ (i.e. the number of tokens sent back to the Investor) based on monetary gain, guilt, and inequity. Two free parameters (theta, Θ; and phi, Φ) determine the influence of these three sources of utility (see Eq. 1 in main text). In this model, the Trustee’s monetary gain is defined as *π*_2_ = (*I* * *M*_2_ − *S*_2_)/(*I* * *M*_2_), where *I* represents the Investor’s investment and *M*_2_ represents the multiplier known only to the Trustee. Inequity is defined *Inequity*_2_ = ((*I* * M_2_  − *S*_2_)/(10 − *I* + I * M_2_) − ½)^5,10^, and guilt is defined *Guilt*_2_ = ((*E*_2_(*E*_1_(*S*_2_)) − *S*_2_)/*(E*_1_(*M*_1_) * I))^2^^8,10,50^, where *E*_2_(*E*_1_(*S*_2_)) represents the Trustee’s belief about the Investor’s expectations of the Trustee’s strategy and *E*_1_(*M*_1_) represents the Investor’s belief about the multiplier. To maximize generalizability of the model, we set the Trustee’s belief about the Investor’s expectations to half of the amount the Investor believes the Trustee has: *E*_2_(*E*_1_(*S*_2_)) = ½ * E_1_(*M*_1_) * *I* (for validation, see^10^)*.* At each reciprocity decision, either guilt or inequity constitutes the social preference term of the Trustee’s utility function. By default, at Φ = 0, the model selects the weakest of the two. This structure explicitly accommodates the ‘moral opportunism’ behavioral pattern, since it allows the Trustee to disregard guilt (i.e. disappointing the Investor) in the lowered-multiplier context (e.g. × 2 in the × 2/× 4/× 6 condition), and to disregard inequity in the raised-multiplier context. As phi moves away from 0, however, the model’s behavioral output becomes biased towards the guilt aversion (phi < 0) or inequity aversion (phi > 0) prediction. Higher levels of theta (Θ) bias behavior towards greed. At different combinations of theta and phi, therefore, the model can accommodate guilt aversion, inequity aversion, moral opportunism, and greed.

In this experiment, we compared the performance of the moral strategy model to that of simpler and previously proposed alternative models: greed (Eq. 2), inequity aversion (Eq. 3), and guilt aversion (Eq. 4). In these models, monetary payoff, guilt, and inequity are defined as in the moral strategy model.

2 $$U_{2} \left( {S_{2} } \right) = \pi_{2}$$

3 $$U_{2} \left( {S_{2} } \right) = \pi_{2} {-}\Theta *Inequity_{2}$$

4 $$U_{2} \left( {S_{2} } \right) = \pi_{2} {-}\Theta *Guilt_{2}$$

Across all models, the predicted strategy for the Trustee was the strategy which yielded maximal utility:

5 $$\hat{S}_{2} = \mathop {\text{arg max}}\limits_{{S_{2} }} U_{2} \left( {S_{2} } \right)$$

Model performance was measured and compared using the Akaike Information Criterion (AIC; Eq. 6)^51,52^, which rewards model fit and penalizes model complexity (number of free parameters). We chose to use AIC over the alternative Bayesian Information Criterion, since AIC is superior to BIC if the true data-generating model is not in the model set^53^, which is likely true for the current experiment. Assuming that the model errors are normally distributed, AIC is defined as

6 $$AIC = n*\ln \left( {SSE{/}n} \right) + k*2$$

where *SSE* represents the residual sum of squares (i.e. the sum over squared differences between model prediction and actual behavior), *n* represents the number of observations (trials), and *k* represents the number of free parameters in the model (theta and/or phi). Participants whose behavior was near-perfectly explained by any model (SSE < 10) were excluded from model comparisons, since the logarithm of 0 is undefined.

To avoid obtaining a local rather than global optimum, the computational models were fit to each participant’s behavior 1000 times, each time with a different random parameter set established as the starting point for the optimizer. For each participant, the best-fitting parameter set from of all solutions was selected.

We used the moral strategy model results to classify participants as greedy, guilt-averse, inequity-averse, or morally opportunistic respectively, using a similar procedure as in earlier work^10^. To this end, we first ran simulations of the moral strategy model for all combinations of investment and multiplier at 10,201 (101 by 101) equidistant points in the model’s theta–phi parameter space (theta on the domain [0, 0.5] and phi on [− 0.1, 0.1]), and applied hierarchical clustering to these simulations based on their pairwise Euclidean distance in reciprocity behavior. In the simulations, we used as second-order expectations half of the amount the Investor believes the Trustee possesses. For the × 2/× 4/× 6 block, we set the clustering distance cutoff such that five clusters were obtained, and then merged the two clusters whose simulations corresponded to the theoretical moral opportunism pattern. For the × 4/× 6/× 8 context, we set the distance cutoff such that four clusters were immediately obtained. This method ensured that high phi corresponded to inequity aversion, low phi to guilt aversion, phi around zero to moral opportunism, and high theta to greed, all of which are line with the design of the moral strategy model, while letting the clustering algorithm draw the precise boundaries between the corresponding zones of theta–phi coordinates in the model’s parameter space. The clustering solution was thus not based on the participant behavior that occurred in our sample, but instead on the theoretical range of behavior that the model could capture. We then located each participant in the parameter space by their best-fitting theta–phi parameter pair, and thus placed them into one of the four moral strategy zones. This procedure yielded, for each participant and each block, a moral strategy label: inequity-averse, guilt-averse, morally opportunistic, and greedy. Importantly, this clustering method precisely mirrored the method applied in our neuroimaging study on the Hidden Multiplier Trust Game^10^, as well as the method applied in study 2 here, which means the relative prevalence of the four reciprocity motives can be directly compared across studies.

#### Software

Stimuli were presented on a Windows pc running PsychToolBox 3.0.11 (www.psychtoolbox.org) for Matlab 2016a (Mathworks, Natick, MA, USA). Questionnaire data, screening, and debriefing were collected using Castor Electronic Data Capture (www.castoredc.com). Computational model analysis was carried out in Python 2.7, where models were fit to data using the *optimize.least_squares* function in the *Scipy* package version 1.0.0^54^.

### Study 2

#### Participants

57 participants were recruited from the Nijmegen student body using an online recruitment tool. Exclusion criteria were identical to those of study 1 and 2 participants were excluded due to disbelief in the task, leaving 55 participants (14 men and 41 women; mean age 21.8 ± 2.8 years).

#### Task

In this study, participants played an ‘inverted’ version of the Hidden Multiplier Trust Game, the False-Belief Multiplier Trust Game (FBMTG), always in the role of Trustee. In this variant, the multiplier by which the Investment is increased is always × 4, but now the *Investors* have varying beliefs about the multiplier. 50% of the 80 Investors encountered by the Trustee (in single-shot interactions) believed their investment would be multiplied by × 4, 25% believe in × 2, and 25% in × 6. The Trustee knows this, and also knows the multiplier is actually always × 4. As in study 1, in 50% of trials, the number of tokens received by the Trustee differs from the number of tokens the Investor believes the Trustee has, which means the reciprocity behavior predicted by the guilt aversion and inequity aversion models again diverges. However, whereas in study 1 (and the original HMTG design) the number of tokens sent to the Trustee changes across conditions, in study 2 the number of tokens the Investor believes the Trustee to have changes across conditions. As in study 1, the FBMTG design also allows for morally opportunistic behavior: a morally opportunistic Trustee could return the Investor’s expectation in × 2 (GA), keeping the surplus; and could create an even split in × 6 (IA), thereby disappointing the Investor.

#### Procedure

After arriving at the laboratory, the participants were seated in a closed-off behavioral lab space, where they were screened for participation and gave written, informed, consent. They then were presented with the general task instructions. The participants were informed that anonymous other participants (‘Player A’) had made ‘Investment Game’ investments in them under varying multipliers (× 2, × 4 and × 6). They learned that they would respond to these investments in the role of Trustee (‘Player B’) and would be free to choose the number of tokens they wished to send back to each anonymous investor. They were also informed that one of the 80 interactions would be randomly selected to be actually paid out to them and the corresponding Investor. In reality, the investment decisions were pre-programmed identically to study 1, and the selected trial was only financially consequential for the Trustee participant.

After reading these instructions, the participants reported how many of 10 game tokens they would invest if they were the Investor, under each of the three multipliers × 2, × 4 and × 6. For each of these investments, they also reported how many tokens they would expect back from an anonymous Trustee. Next, as in study 1, we asked permission to take the participant’s portrait photo and use this alongside their investment(s) in a potential future study. Finally, the participants were given a page with extra instructions for this experiment, which stated that although the Investors believed in varying multipliers (× 2, × 4 and × 6), the multiplier would in fact always be × 4. The participants were quizzed on these instructions, and once they understood started playing 80 rounds of the FBMTG.

On each game round, the participants were presented with the Investor’s participant number and a blurred photo of their face, as in study 1. They next read how much the Investor had invested in them, and by how much the Investor believed their investment would be multiplied. They were also reminded that the investment would in fact be multiplied by 4. On the final task screen, the participants indicated how many of the multiplied game tokens they wished to return to the Investor, with the option of keeping all. Halfway through the task, i.e. after 40 trials, the participants could take a (self-paced) break.

#### Additional measures

After the main task, we collected the same additional measures as in study 1: the participants completed an extensive questionnaire about their second-order expectations (i.e. beliefs about the Investor’s expectations) and fairness norms in the FBMTG; they completed a computerized version of the Social Value Orientation ‘slider measure’ task; and filled out the Guilt Inventory, the Dispositional Greed Scale, and a debriefing questionnaire. In this final step, they self-reported how they made their decisions in the main task by dividing 100 points over various candidate strategies (see study 1). The same measures as in study 1 were taken to ensure and check that the participants believed the Investors were real. Two participants indicated that they did not believe the Investors were real other participants; their data were excluded from analysis.

## Supplementary information

Supplementary Figure.

Supplementary Legend.

## Data Availability

All data and code necessary to reproduce the results in this paper are available at https://github.com/jeroenvanbaar/ReciprocityMotives.
