# Supplementary material for: Stable distribution of reciprocity motives in a population
Source: Sci Rep. 2020 Oct 23;10:18164. doi: 10.1038/s41598-020-74818-y (PMC7584663; doi:10.1038/s41598-020-74818-y)
Supplement: Supplementary file 1 — Supplementary Figure. [file 41598_2020_74818_MOESM1_ESM.pdf]

Supplemental Information for

Stable distribution of moral strategies in a population

Jeroen M. van Baar[1,2]*, Felix Klaassen[2], Filippo Ricci[2,3], Luke J. Chang[4], Alan G. Sanfey[2,5]

[1] Department of Cognitive, Linguistic, and Psychological Sciences, Brown University, United States
[2] Donders Institute for Brain, Cognition, and Behavior, Radboud University, The Netherlands
[3] Utrecht University School of Economics, Utrecht University, The Netherlands
[4] Department of Psychological and Brain Sciences, Dartmouth College, United States
[5] Behavioral Science Institute, Radboud University, The Netherlands
* Correspondence to: jvb@brown.edu

This PDF contains:
Supplemental Figure 1, panels A-L (pages 1-12)

Supplemental Figure 1
A

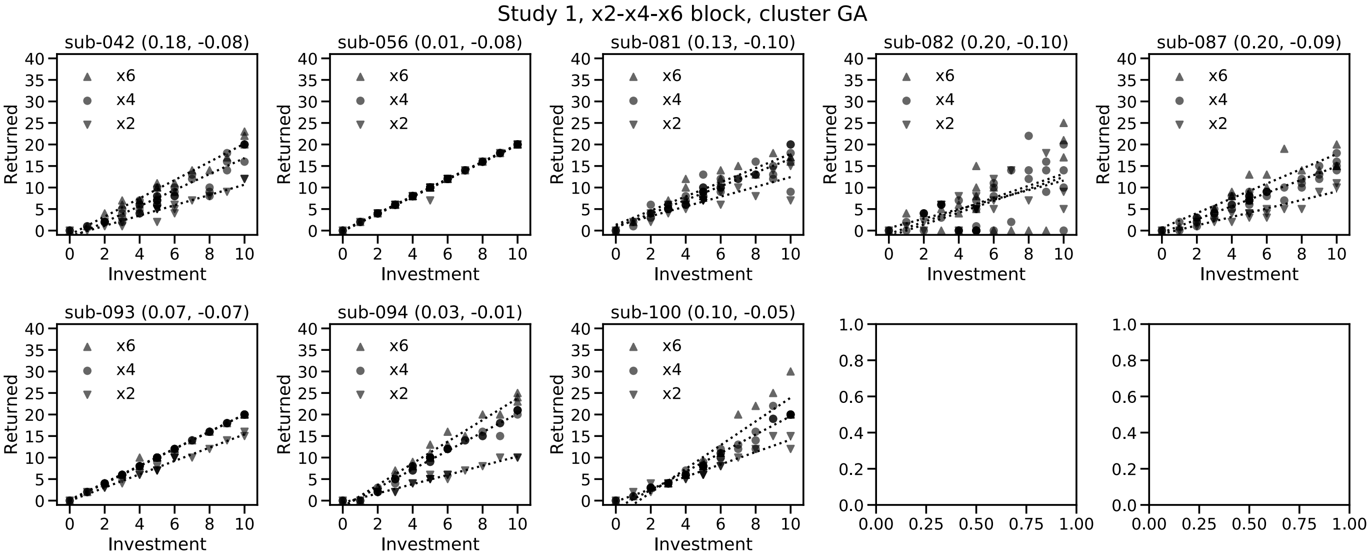

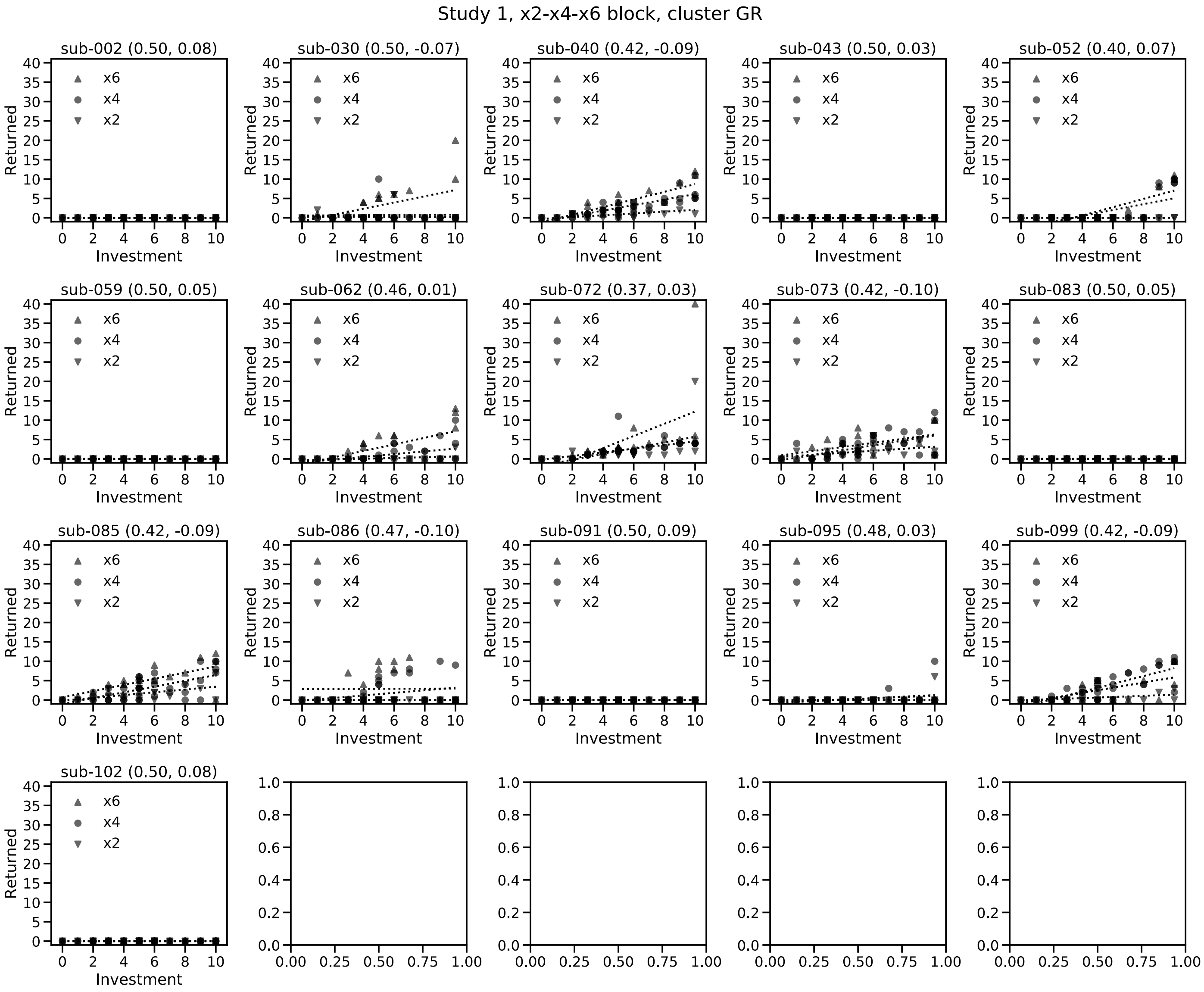
Study 1, x2-x4-x6 block, cluster GR
sub-002 (0.50, 0.08)
sub-030 (0.50, -0.07)
sub-040 (0.42, -0.09)
sub-043 (0.50, 0.03)
sub-052 (0.40, 0.07)
sub-059 (0.50, 0.05)
sub-062 (0.46, 0.01)
sub-072 (0.37, 0.03)
sub-073 (0.42, -0.10)
sub-083 (0.50, 0.05)
sub-085 (0.42, -0.09)
sub-086 (0.47, -0.10)
sub-091 (0.50, 0.09)
sub-095 (0.48, 0.03)
sub-099 (0.42, -0.09)
sub-102 (0.50, 0.08)
x6
x4
x2
Returned
Investment

B

Study 1, x2-x4-x6 block, cluster IA

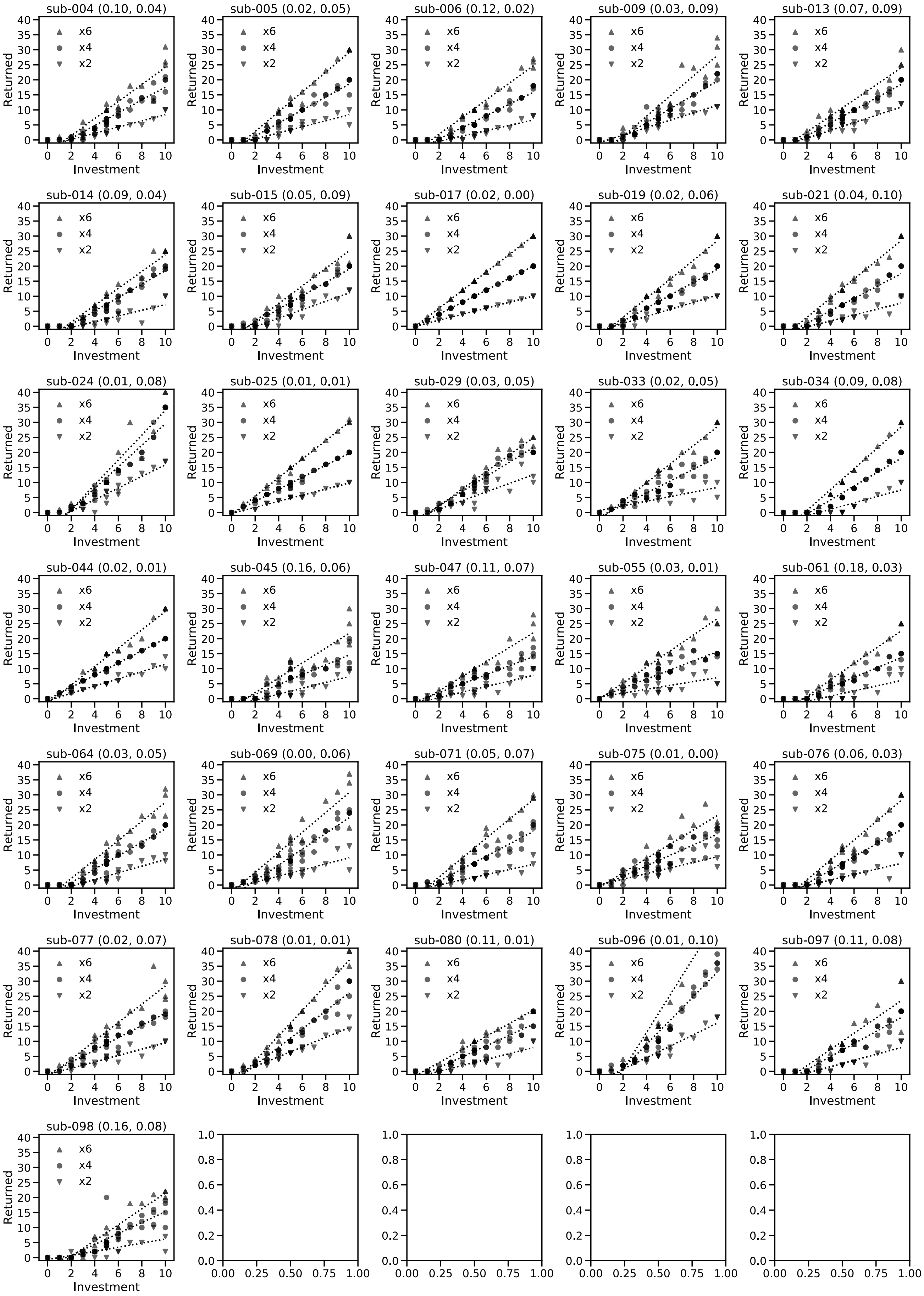

C

Study 1, x2-x4-x6 block, cluster MO

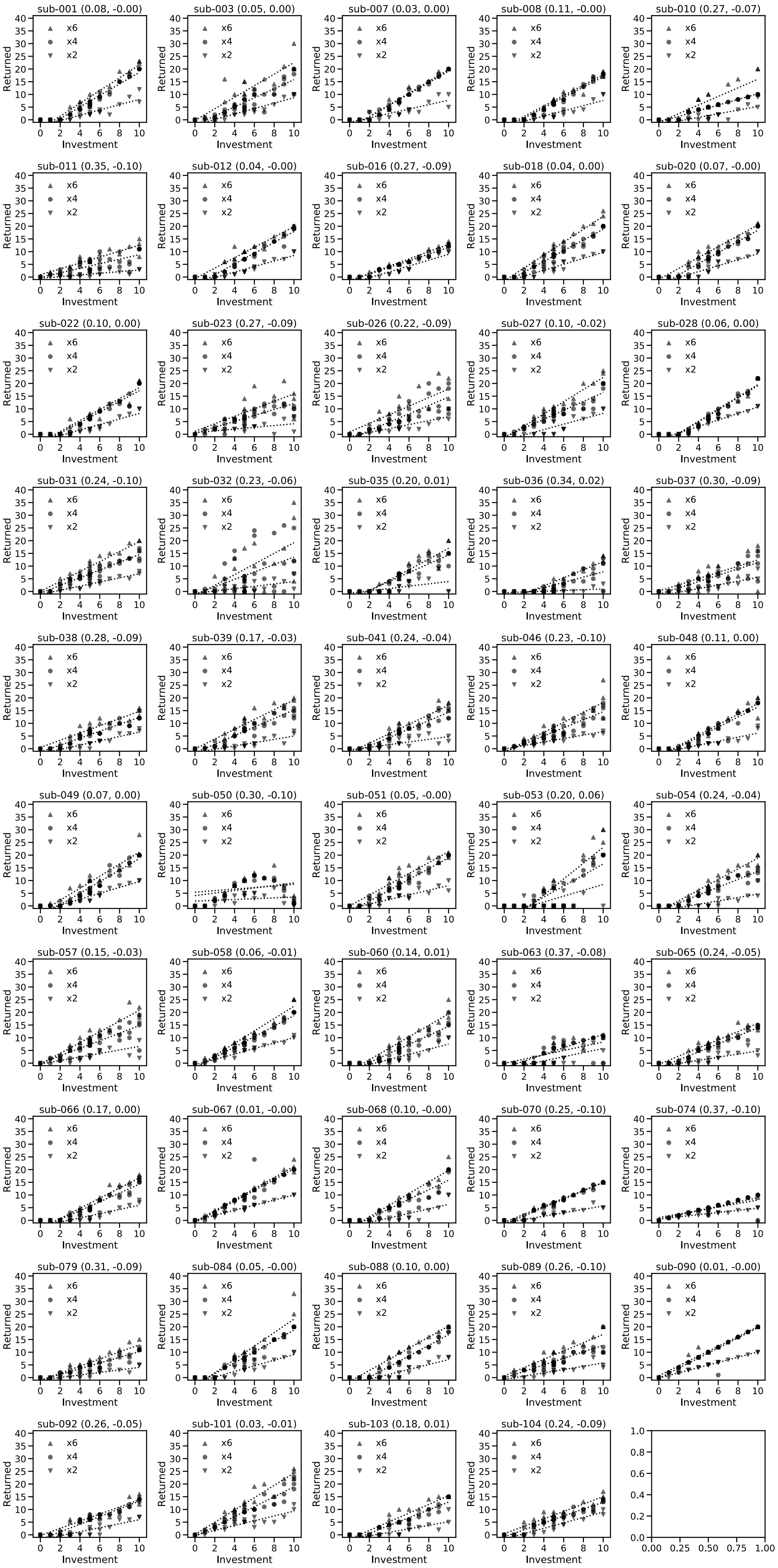

D

Study 1, x4-x6-x8 block, cluster GA

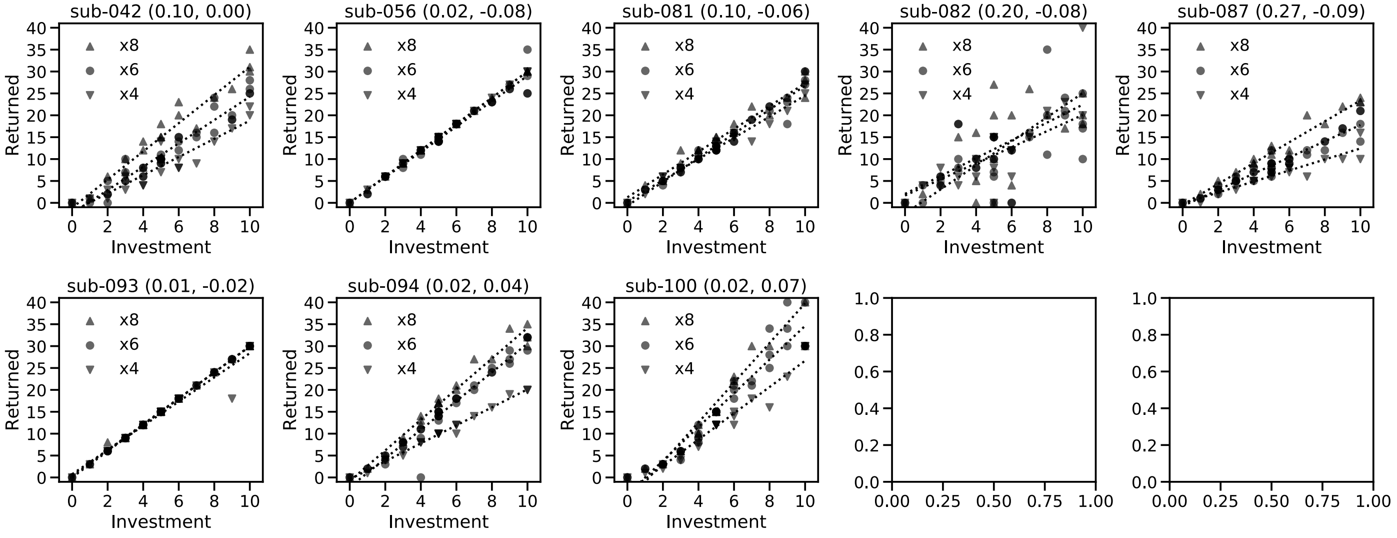

E

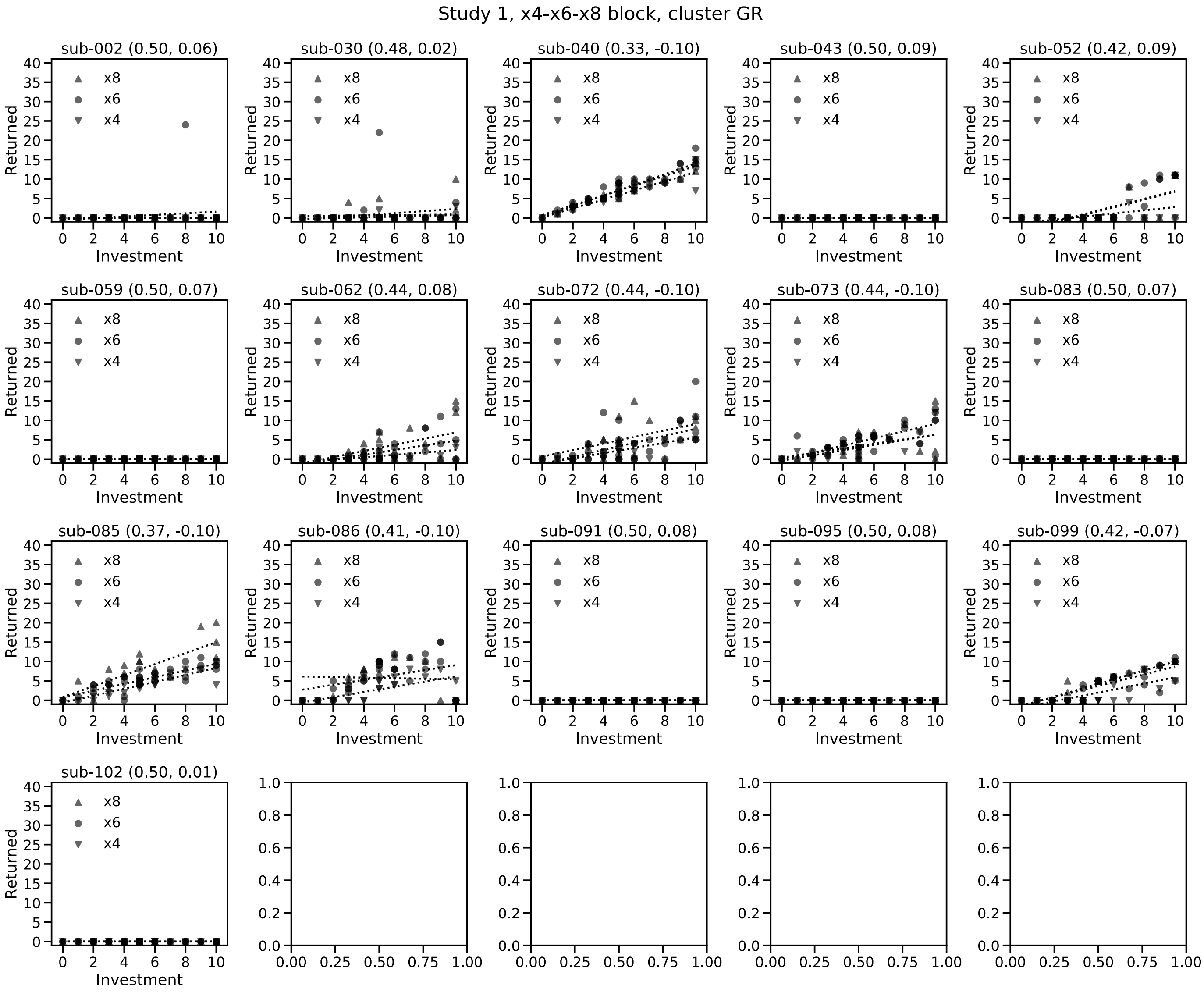
Study 1, x4-x6-x8 block, cluster GR
sub-002 (0.50, 0.06)
sub-030 (0.48, 0.02)
sub-040 (0.33, -0.10)
sub-043 (0.50, 0.09)
sub-052 (0.42, 0.09)
sub-059 (0.50, 0.07)
sub-062 (0.44, 0.08)
sub-072 (0.44, -0.10)
sub-073 (0.44, -0.10)
sub-083 (0.50, 0.07)
sub-085 (0.37, -0.10)
sub-086 (0.41, -0.10)
sub-091 (0.50, 0.08)
sub-095 (0.50, 0.08)
sub-099 (0.42, -0.07)
sub-102 (0.50, 0.01)
x8
x6
x4
Returned
Investment

F

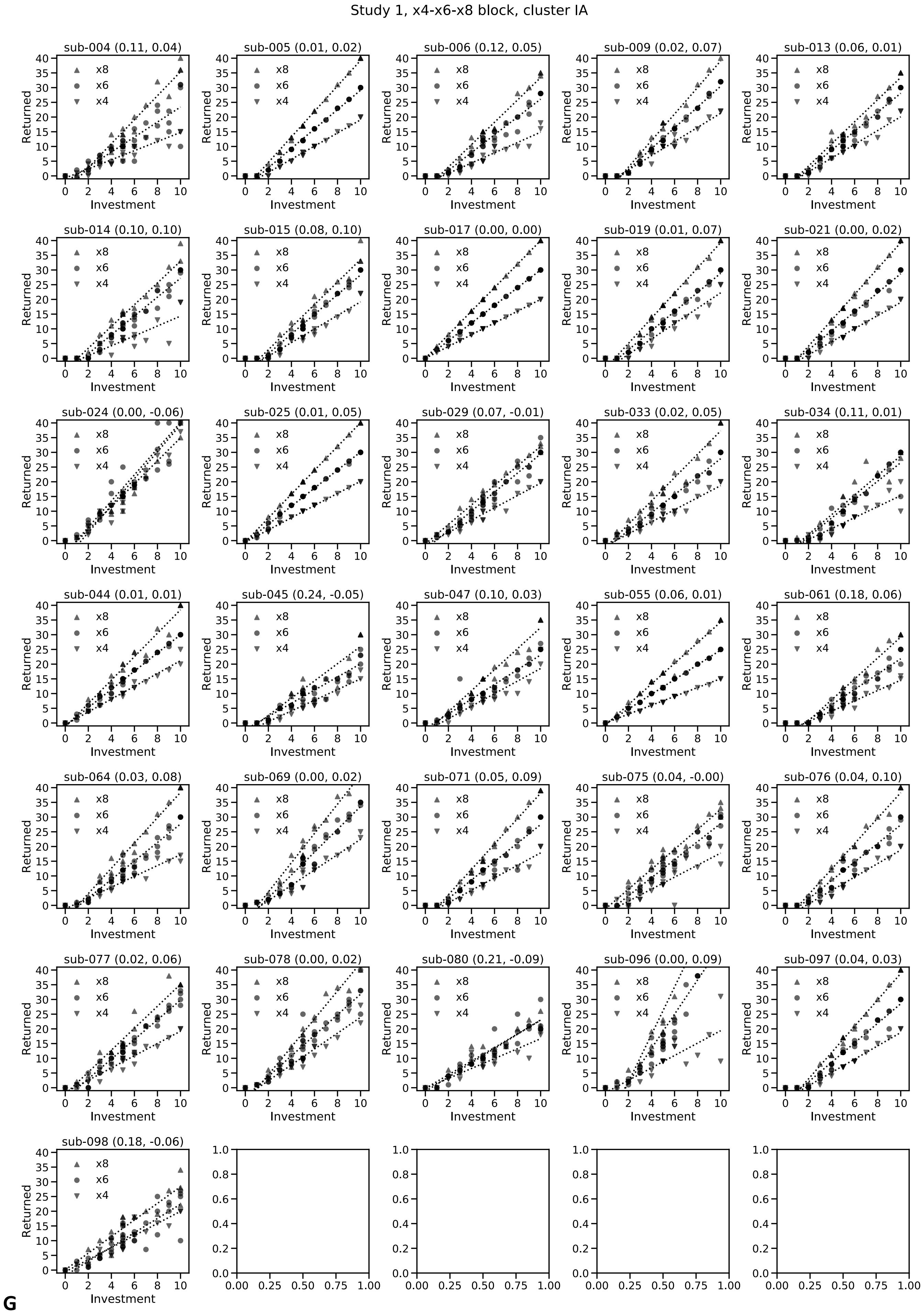
Study 1, x4-x6-x8 block, cluster IA
sub-004 (0.11, 0.04)
sub-005 (0.01, 0.02)
sub-006 (0.12, 0.05)
sub-009 (0.02, 0.07)
sub-013 (0.06, 0.01)
sub-014 (0.10, 0.10)
sub-015 (0.08, 0.10)
sub-017 (0.00, 0.00)
sub-019 (0.01, 0.07)
sub-021 (0.00, 0.02)
sub-024 (0.00, -0.06)
sub-025 (0.01, 0.05)
sub-029 (0.07, -0.01)
sub-033 (0.02, 0.05)
sub-034 (0.11, 0.01)
sub-044 (0.01, 0.01)
sub-045 (0.24, -0.05)
sub-047 (0.10, 0.03)
sub-055 (0.06, 0.01)
sub-061 (0.18, 0.06)
sub-064 (0.03, 0.08)
sub-069 (0.00, 0.02)
sub-071 (0.05, 0.09)
sub-075 (0.04, -0.00)
sub-076 (0.04, 0.10)
sub-077 (0.02, 0.06)
sub-078 (0.00, 0.02)
sub-080 (0.21, -0.09)
sub-096 (0.00, 0.09)
sub-097 (0.04, 0.03)
sub-098 (0.18, -0.06)
x8
x6
x4
Returned
Investment
G

Study 1, x4-x6-x8 block, cluster MO

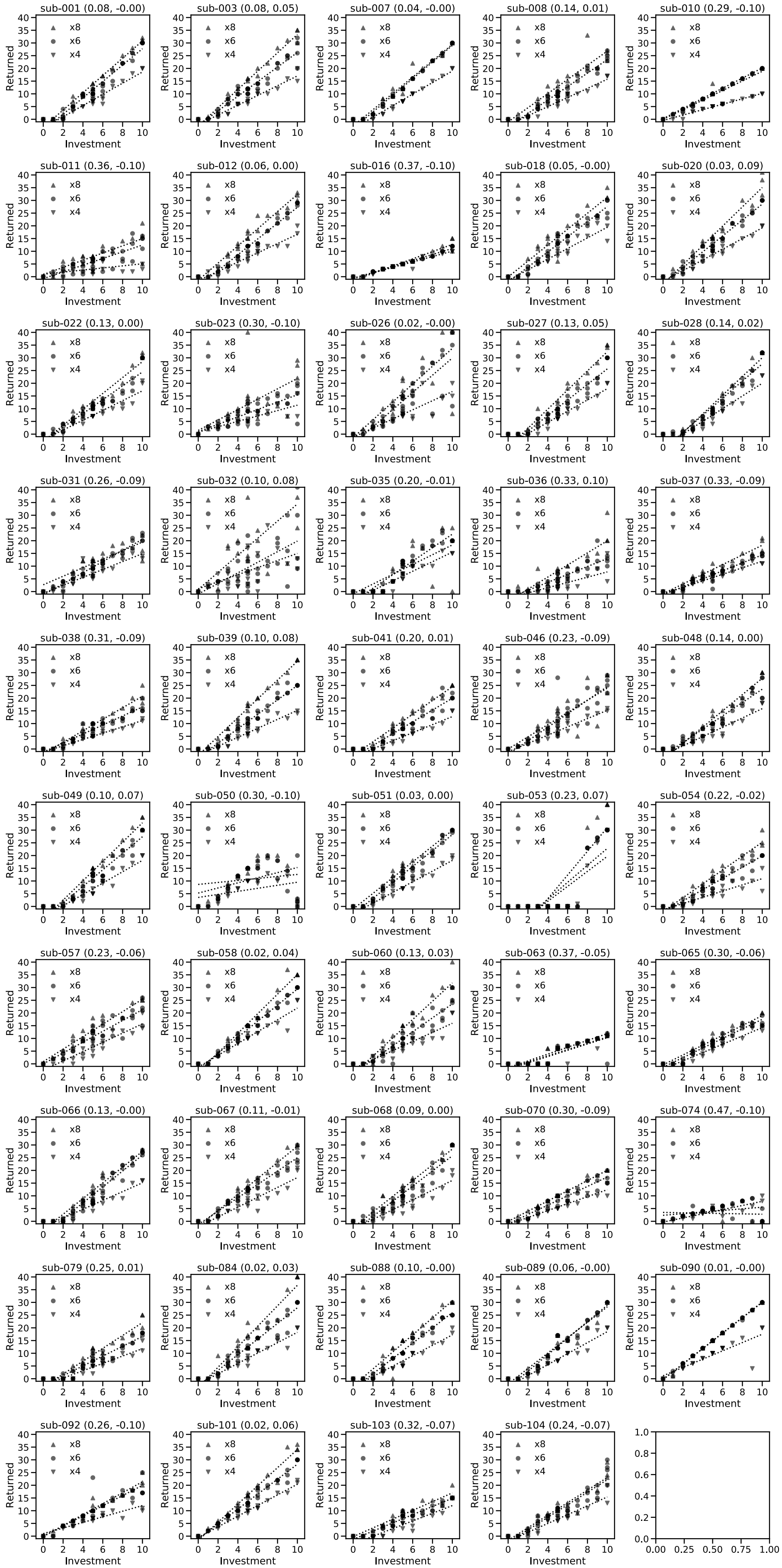

H

Study 2, cluster GA

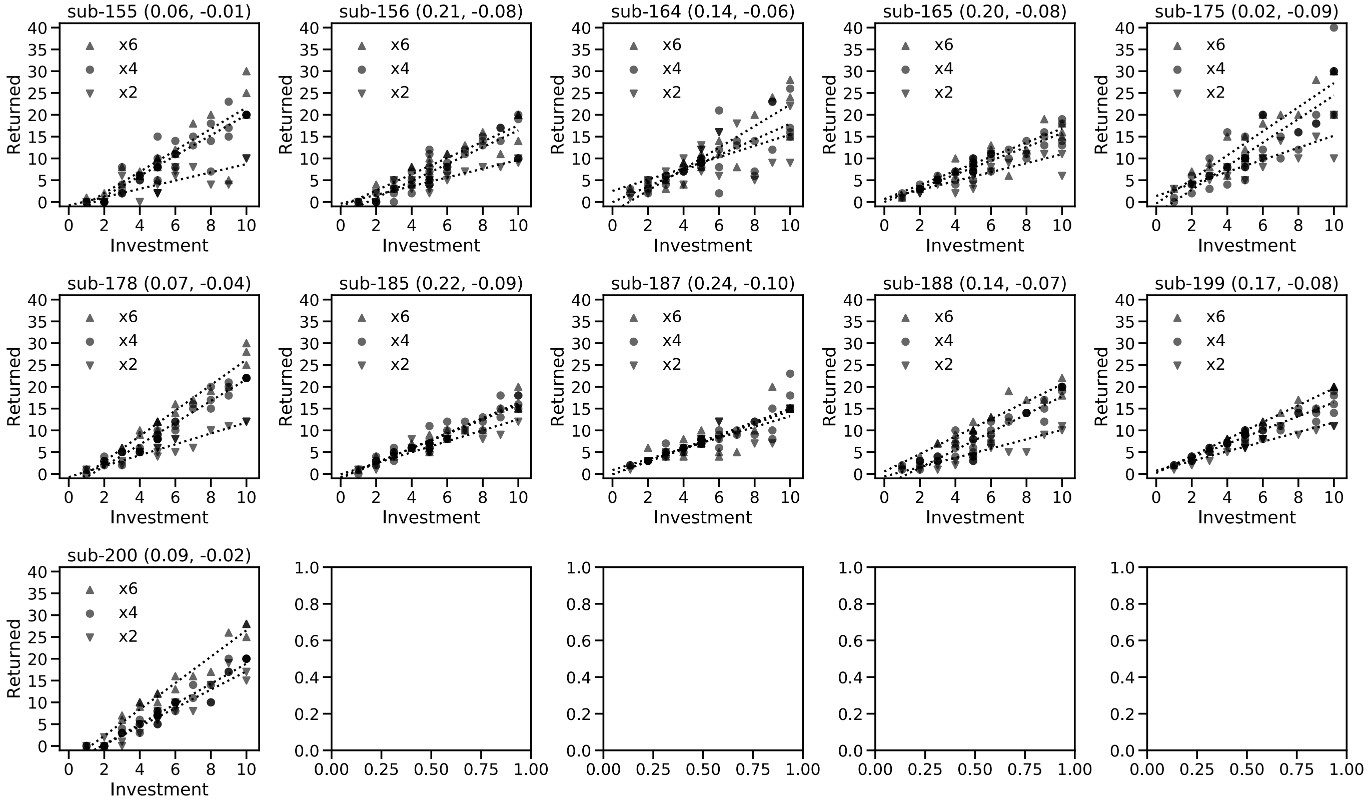

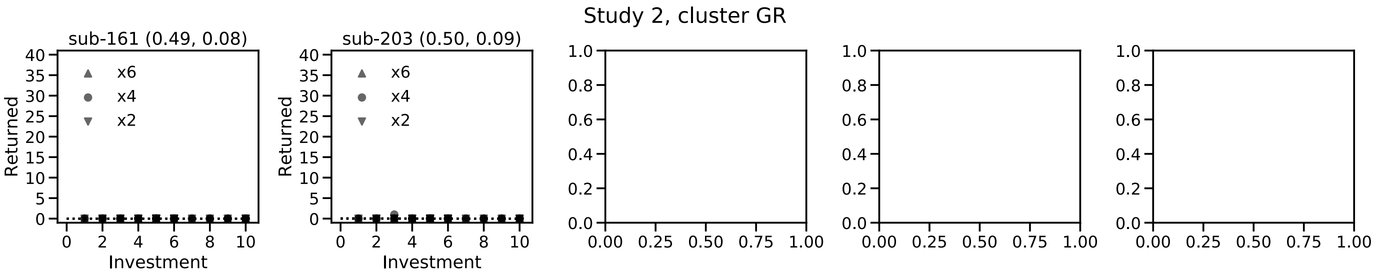
Study 2, cluster GR
sub-161 (0.49, 0.08)
sub-203 (0.50, 0.09)
x6
x4
x2
Returned
Investment

J

Study 2, cluster IA

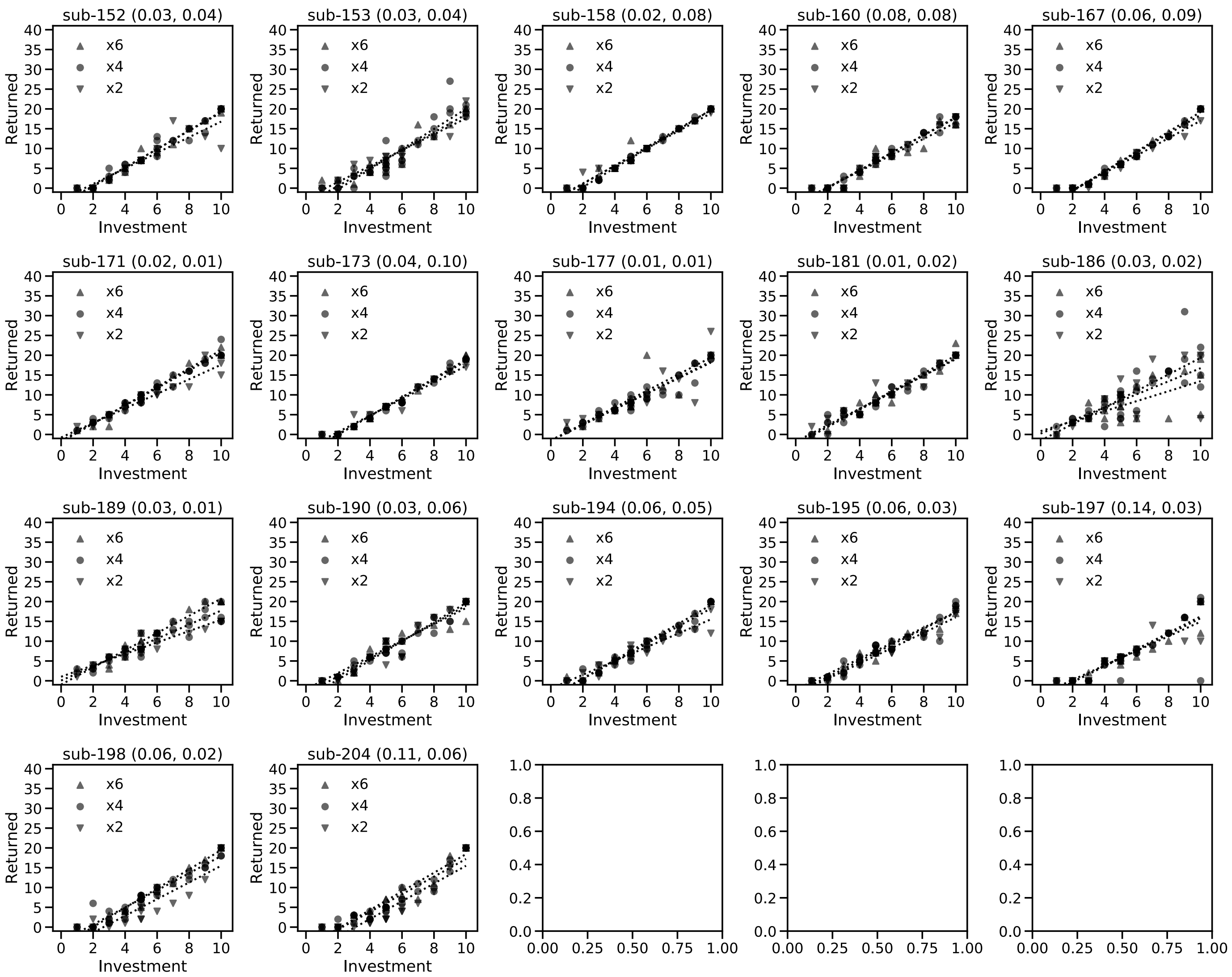

K

Study 2, cluster MO

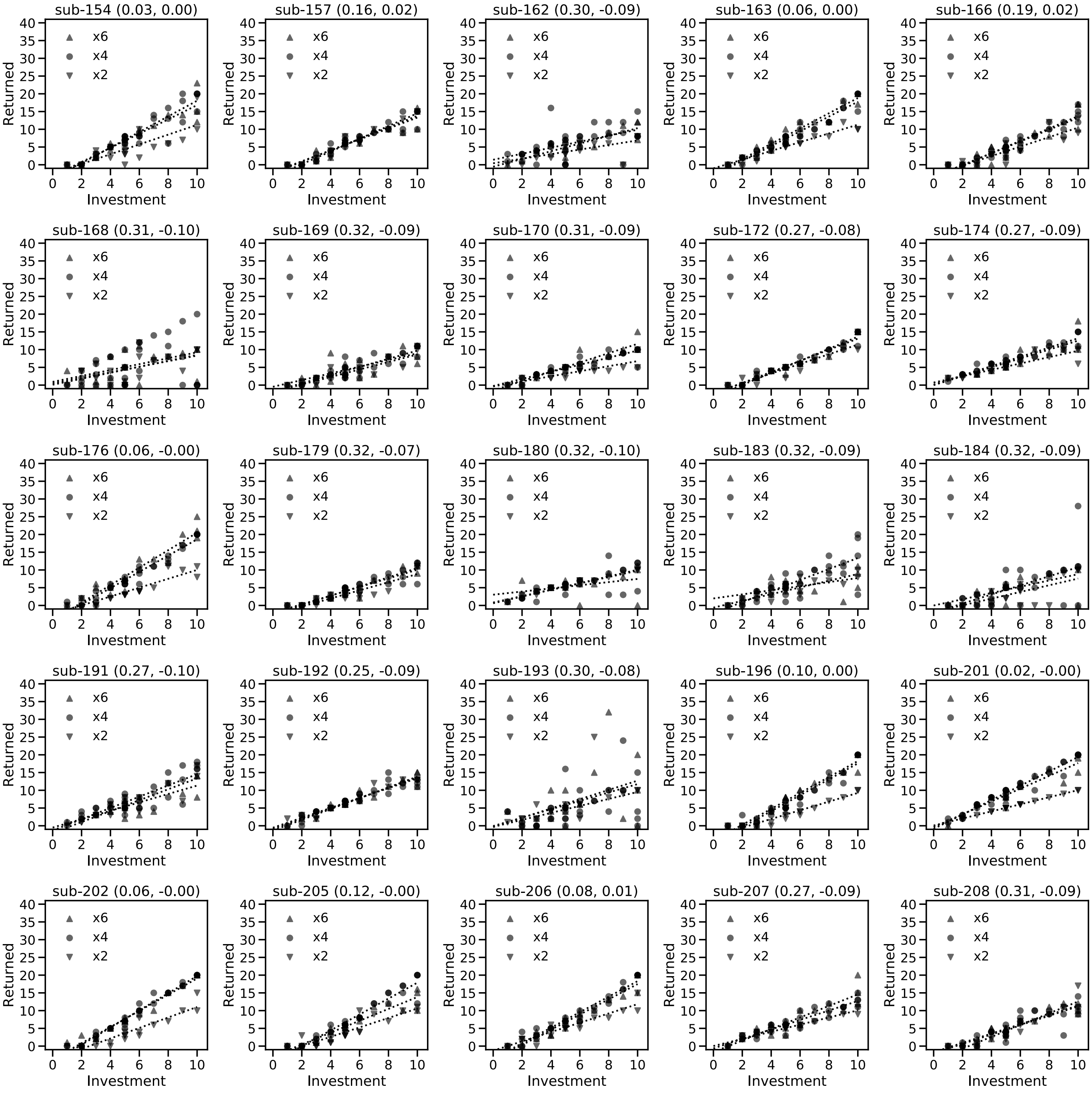

L
