# Supplementary material for: Stable distribution of reciprocity motives in a population
Source: Sci Rep. 2020 Oct 23;10:18164. doi: 10.1038/s41598-020-74818-y (PMC7584663; doi:10.1038/s41598-020-74818-y)
Supplement: Supplementary file 2 — Supplementary Legend. [file 41598_2020_74818_MOESM2_ESM.docx]

**Supplemental Figure Legends**

*Supplementary Figure 1. Task behavior of all participants, grouped by moral strategy cluster as derived from the computational model. A-D: Study 1, x2/x4/x6 block. E-H: Study 1, x4/x6/x8 block. I-L: Study 2.*
